# Review on the Properties of Boron-Doped Diamond and One-Dimensional-Metal-Oxide Based P-N Heterojunction

**DOI:** 10.3390/molecules26010071

**Published:** 2020-12-25

**Authors:** Yu Yao, Dandan Sang, Susu Duan, Qinglin Wang, Cailong Liu

**Affiliations:** Shandong Key Laboratory of Optical Communication Science and Technology, School of Physics Science and Information Technology, Liaocheng University, Shandong 252000, China; lcuyaoyu0814@163.com (Y.Y.); dss1039860962@163.com (S.D.)

**Keywords:** diamond-based, one-dimensional metal oxide, heterojunction, high temperature

## Abstract

This review is mainly focused on the optoelectronic properties of diamond-based one-dimensional-metal-oxide heterojunction. First, we briefly introduce the research progress on one-dimensional (1D)-metal-oxide heterojunctions and the features of the p-type boron-doped diamond (BDD) film; then, we discuss the use of three oxide types (ZnO, TiO_2_ and WO_3_) in diamond-based-1D-metal-oxide heterojunctions, including fabrication, epitaxial growth, photocatalytic properties, electrical transport behavior and negative differential resistance behavior, especially at higher temperatures. Finally, we discuss the challenges and future trends in this research area. The discussed results of about 10 years’ research on high-performance diamond-based heterojunctions will contribute to the further development of photoelectric nano-devices for high-temperature and high-power applications.

## 1. Introduction

Metal oxide materials have attracted great attention from the scientific community because of their important technical applications. One-dimensional (1D) nanorods (NRs), nanowires (NWs) and nanotubes (NTs) formed from metal oxides (such as ZnO, TiO_2_ and WO_3_) enable fabrication of some specific nanodevices for optoelectronic applications, for instance, photodetectors [1,2], light-emitting diodes (LED) [3], and solar cells [4,5]. This is because of the large surface-area-to-volume ratio, excellent charge carrier transport performance and good crystallization ability shown by these types of nanostructures [6,7]. Thus far, heterostructure optoelectronic devices have been formed by depositing n-type metal oxides on various p-type substrates, including Si [8,9,10,11], GaN [12,13,14], NiO [15], Cu_2_O [16,17,18,19], graphene [20,21], boron-doped diamond (BDD) film [22,23,24,25,26,27], and organic material [28]. Among them, BDD acts as an excellent p-type conductive material for high-temperature, high-power and radiation-proof photoelectronic devices with its large band gap at room temperature (5.47 eV) and high thermal conductivity [29]. When combining 1D metal oxide with p-type diamond, one has to explore the carrier transport behavior of the formed heterojunction devices, which has both theoretical and application importance for designing new photoelectronic devices for extremely harsh environments, such as outer space or nuclear energetics industries. In recent years, p-type BDD has been used in combination with various 1D-structured metal oxides (for instance ZnO [22,30,31,32,33], WO_3_ [34,35,36] and TiO_2_ [37,38,39,40,41]) to form heterojunctions demonstrating effects of rectification and negative differential resistance (NDR), which may be widely used in various technologies. However, no comprehensive discussion focusing specifically on electrical characteristic of diamond-based p-n heterojunctions has been published. Therefore, this review summarizes the past progress in the device fabrication, electrical transport properties and NDR-related applications of diamond-metal oxides (ZnO, TiO_2_ and WO_3_) heterojunctions. The temperature-driven carrier injection mechanisms in such heterojunctions are described herein in further detail. The approximately 10 years of research discussed will benefit the next step in the development of high-temperature and high-power optoelectronic devices.

## 2. Heterojunctions with 1D Metal Oxides Semiconductors

Since the discovery of carbon NTs by Professor Iijima in 1991 [42], 1D nanomaterials have quickly become one of the hotspots in the research of nanomaterials and functional devices. Carbon NTs have excellent physical and chemical properties, such as large surface-area-to-volume ratio, high mechanical strength and brilliant thermal conductivity, as well as good chemical stability. However, the use of presently existing growth technologies does not allow a readily available effective chiral control over the synthesis of carbon NTs [43], always resulting in a compound with both metallic and semiconducting properties. As such, it is impossible to obtain a completely pure semiconductor, which greatly limits the applications of optoelectronic devices.

Because of the above limitation, researchers started with the development and investigation of other 1D semiconductor nanomaterials. Silicon (Si) is the most widely used semiconductor material for optoelectronics devices. Professor Charles Lieber of Harvard University, as the leading expert in this field, for the first time has successfully prepared Si NWs [44] and employed them in photovoltaic sub-devices, biosensors, etc. [45,46], showing the revealed nanomaterials and devices to have broad-range expectations in engineering.

However, Si NWs also demonstrate some shortcomings, such as easy oxidation to form polycrystalline or crystal defects in air, which may have a prevailing effect on the electrical transmission characteristic. Due to this fact, researchers reoriented to the development and implementation of 1D nanometer semiconducting materials with less oxidizing ability and higher stability. In 2001, the discovery of metal oxide semiconductor nanobelts pushed the study of nanomaterials forward to a new challenge. Metal oxides as the prime candidates for new functional inorganic materials are finding many promising applications in aerospace, biological engineering, semiconductor electronics, functional ceramics, and other fields. The 1D metal oxide nanosystems belong to the most prominent examined systems due to their good crystal quality, low defect density, excellent charge-carrier mobility, and fast response [47], thereby forming a promising replacement for traditional silicon-based electronic and optical devices.

**Figure 1:**
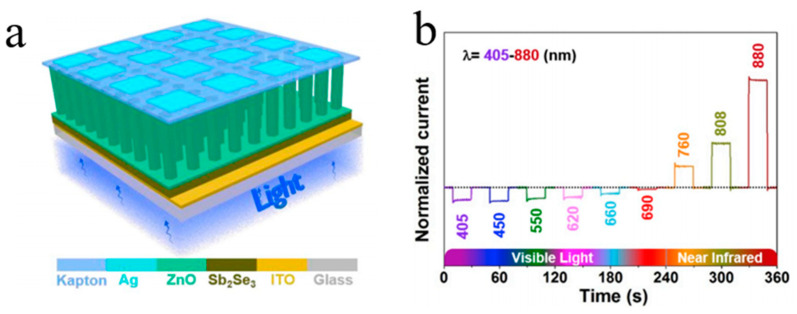
(**a**) Schematic view of the photodetector and (**b**) normalized photocurrent under illumination of light with different wavelengths [49].

The most recent studies have reported on the excellent optical–electronic performance of 1D metal oxides as well as new functional devices based on various substrate materials. Du et al. prepared a high-speed ultraviolet photoelectricity detector of ZnO-NWs Schottky barrier based on the surface-ionic-gate powered by tribo-nanometer generator [48]. Wang et al. demonstrated a novel bipolar response in self-powered ZnO NWs/Sb_2_Se_3_ heterojunction photodetector with adjustable polarity switching wavelength. As demonstrated in Figure 1, the output signal shows the change in the photocurrent polarity between shorter (405 nm–690 nm) and longer (760 nm–880 nm) wavelength regions [49]. Gao et al. have recently obtained a UV-free white LED based on high-level Fe-doped p-ZnO NWs arrays on the n-GaN substrate [12]. Peng et al. developed a real-time wearable UV-radiation monitor by exploiting the excellent properties of p-CuZnS/n-TiO_2_ photodetector [50]. Tang et al. proposed a feasible way to improve the hole doping in ZnO:N films with introduced beryllium and demonstrated strong near-band edge UV emission of the ZnO homojunction LED devices, which can be observed even at 400 K under continuous current injection [51]. Ye et al. improved the performance of n-ZnO NRs/p-GaN LED with the use of transparent graphene electrode. The transparent graphene electrode was used as the current diffusion layer, showing better performance compared to that in the ITO analogs [52]. The photoelectrochemical self-powered photodetectors related to ZnO/CdS NWs were manufactured by Zhang’s group [53]. The ZnO NWs were used as the carrier collection channels and UV absorbers with a well-organized structure to efficiently absorb light, whereas CdS nanoparticles were used as the visible photosensitizers. Prepared ZnO NWs/CdS structures demonstrate superfast response time and effective sensitivity to visible light and UV in the absence of power.

**Figure 2:**
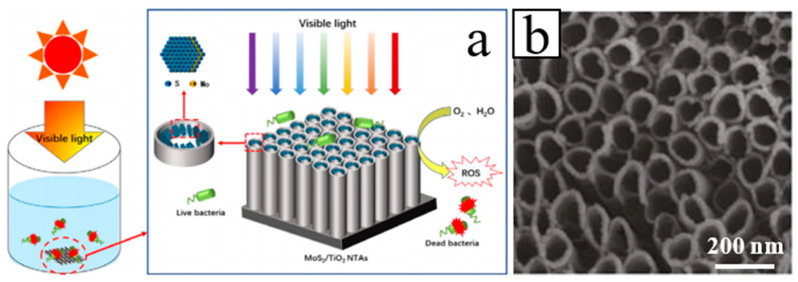
(**a**) Schematic diagram and (**b**) SEM image of the MoS_2_/TiO_2_ NTs fabrication process [55].

Cheng et al. fabricated a SnS/TiO_2_ NTs arrays photoelectrode synthesized by anodization combined with electrodeposition technique, which was used to degrade 2,4,6-trichlorophenol under simulated visible light irradiation [54]. Yan et al. prepared photocatalytic binary composite MoS_2_/TiO_2_ (NTs) heterojunction (Figure 2). The composite material has demonstrated good photocatalytic disinfection effect and recyclability and as such has a broad field of potential applications in water disinfection [55]. Gu et al. reported on the preparation of novel WO_3_ NRs/graphene/BiV_1−x_Mo_x_O_4_ heterojunction photoelectrode for photoelectrochemical water splitting. The heterojunction exhibits an enhanced photocurrent density, which makes light conversion efficiency significantly improved [56].

Specifically, these studies provide new strategies and insights for the fabrication of high-efficiency optoelectronic devices. One may see that most of the existing 1D metal oxide heterojunctions are generally based on Si, GaN and Cu_2_O, etc., and such devices show excellent photoelectric performance under normal environmental conditions. However, due to the small size of nanostructures, both light and injection current will cause a significant thermal effect. Since the thermal conductivity of the listed substrates is low, it leads to the rise in the thermal noise phenomenon and fluctuation of the effective barrier height at a high temperature. With the temperature rise, an increase in the leakage current, a drift of the threshold voltage and enhanced thermal noise degree will affect the sensitivity and reliability of the device. In addition, there is a large thermal coefficient difference between the metal oxides and the Si substrate. The interface between these two materials plays an important role in the device performance: the heterogeneity at the interface will cause the effective barrier height to fluctuate with the reduced performance of a device. This phenomenon is more evident in harsh environments such as high temperature and high flux [57,58,59].

## 3. P-Type B-Doped Diamond Films

Compared with other wide band gap semiconducting substrates (NiO_2_, GaN, SiC, etc.), diamond has favorable intrinsic optoelectronic performance, for instance, high thermal conductivity (22 W·cm^−1^·K^−1^), high carrier mobility (2200 and 1800 cm^2^·V^−1^·s^−1^), high electrical breakdown field (10 MV·cm^−1^) and high saturation velocity (2.7 × 10^−7^ cm·s^−1^) [60,61]. Therefore, diamond is regarded as a suitable material for high power and high temperature to cooperate with metal oxide semiconductors in optoelectronic devices. So far, the main requirements include high-quality epitaxial growth as well as doping. However, because of the contradiction between structural quality and the electrical properties of p-diamond, it is still a challenge to produce high-quality p-type diamond structures. With boron doping, diamond can be transformed from an insulator into a semiconductor or even a superconductor, wherein a boron atom is in the form of a host impurity in the diamond. High-boron doping in the diamond film may enhance its electrical resistivity (up to 10^−3^ Ω·cm order). In order to improve the efficiency of B-doping pursuing high mobility and high crystal quality, many efforts have been implemented.

**Figure 3:**
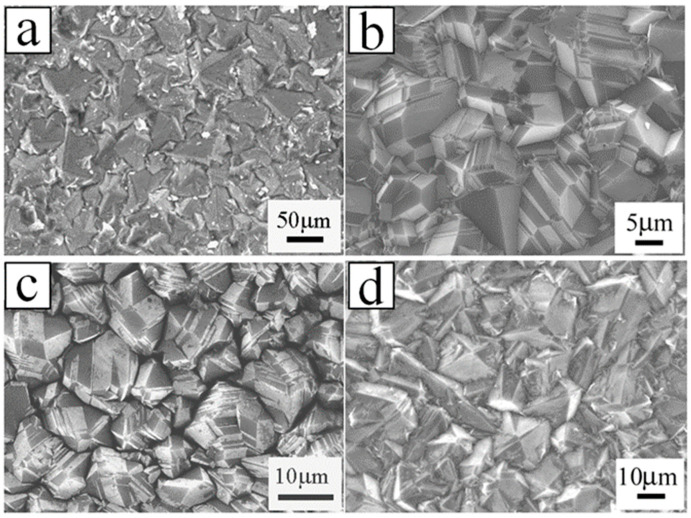
SEM images of the boron-doped diamond (BDD) with plane view grown with the boron of (**a**) 2 sccm, (**b**) 2 sccm, (**c**) 10 sccm and (**d**) 20 sccm [62].

**Figure 4:**
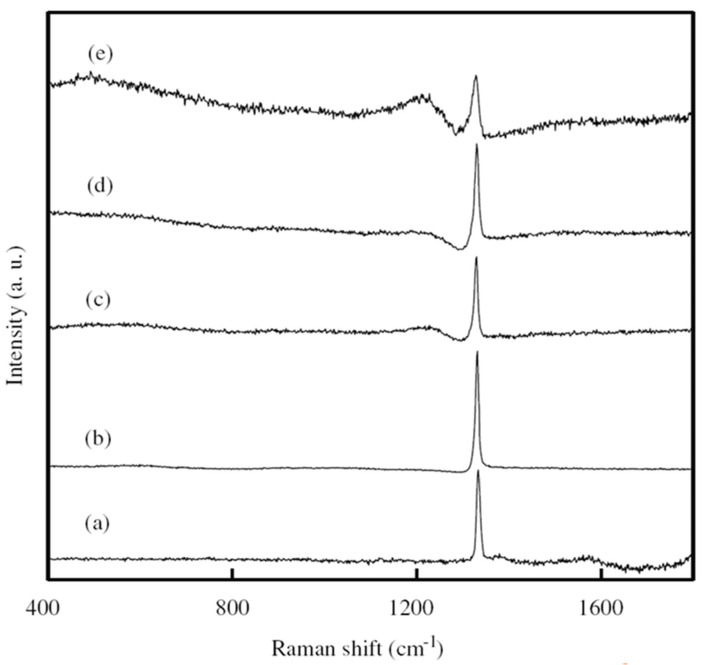
Raman pattern of BDD grown with the boron of (**a**) 0 sccm, (**b**) 2 sccm, (**c**) 5 sccm, (**d**) 10 sccm and (**e**) 20 sccm [62].

Experimentally, Li’s group obtained the BDD film synthesized by hot filament chemical vapor deposition (HFCVD) [62]. B(OCH_3_)_3_ was utilized as the boron doping source in a methane (CH_4_) and hydrogen (H_2_) reaction atmosphere with a flow rate of 0, 2, 5, 10 and 20 sccm. The resistances decrease for the B-doping diamond films grew with increasing the H_2_ flow rate tested by Hall-effect measurement. The undoped diamond film consists of pyramid-shaped grains. With the increase of the B-flow rate up to 2 sccm and 10 sccm, the majority of grains showed lamellar-shaped twin characteristic, on account of the renucleation induced by B-doping. As for 20 sccm, the grains showed a dominating pyramid-shaped morphology, and a twinned crystal appeared (Figure 3). Raman spectroscopy (Figure 4) is an effective technique to investigate the structure of doped diamond. It is worth noting that owing to the high content of BDD, the p-degenerated diamond peak (1332 cm^−1^) shows an asymmetric curve and shifts towards the lower wavelength values in the region-centered phonon bands. Moreover, two wide bands that appear at 500 cm^−1^ and 1200 cm^−1^ in the low-frequency spectrum portion are consistent with two maximum values of phonon density in the diamond state.

**Figure 5:**
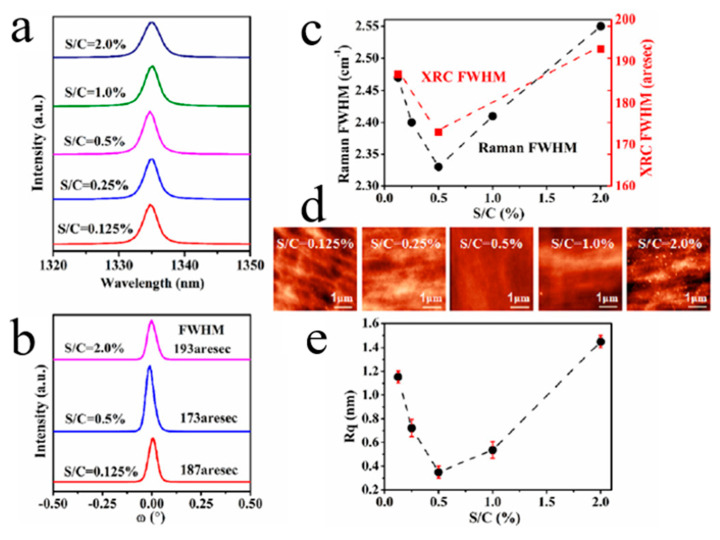
Boron/sulfur co-doped diamond thin films with the different S/C gas proportion: (**a**) Raman pattern. (**b**) X-ray rocking curves (XRCs). (**c**) Full width at half maximum of the Raman pattern and XRCs. (**d**) Atomic force microscopy (AFM) image (5 × 5 μm^2^). (**e**) Root mean square (RMS) surface roughness (Rq) [63].

In the most recent study [63], a small amount of sulfur was added during the deposition of BDD films by microwave plasma chemical vapor deposition (MPCVD), as reported by Liu’s group. The results show the highest values of at once doping efficiency, growth rate, hole mobility and concentration, crystal mass and surface morphology of boron attained with the addition of sulfur (Figure 5). In the presence of an appropriate amount of sulfur, a high carrier concentration of 1.2 × 10^19^ at/cm^3^ may be obtained during the growth process when the B-C ratio is only 2.5 ppm, which denotes a high efficiency of boron doping. The regulation mechanism of sulfur addition has been considered in terms of sulfur-induced plasma changes and possible boron-sulfur complex formation.

**Figure 6:**
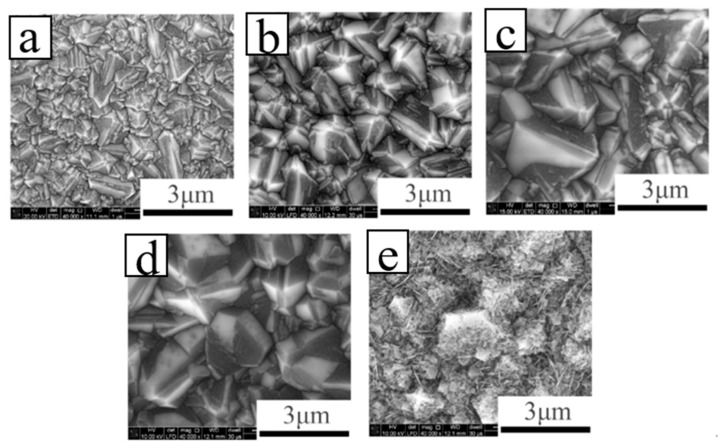
SEM images of BDD films grown with flow rate: (**a**) B 0 sccm, (**b**) B 0.5 sccm, (**c**) B 1 sccm, (**d**) B 2 sccm, (**e**) B 5 sccm [64].

Wei et al. [64] reported on the B-doped double-layer diamond films fabricated by MPCVD and discussed the influence of B-doping concentrations on the surface morphology, crystal quality, surface composition, conductivity and secondary electron emission properties. With increasing boron doping amount, the conductivity becomes beneficial to the emission of secondary electrons. However, as a consequence of the declining quality of the diamond crystals (Figure 6), the increased *sp*^2^ carbon on the surface and the boron segregation on the surface will reduce the effects of secondary electron emission in the films. Therefore, this improves vertical conductance, which increases the escape depth of secondary electrons and obviously leads to the reduced surface performance with the B doping. The results show that the films with the boron-doped layer demonstrate a low concentration of the crystals with excellent quality and sufficient conductivity, which helps to attain the outstanding properties of the secondary electron emission.

Specifically, the discussed results referring to BDD demonstrate strategies for improving the doping efficiency, hole mobility, carrier concentrations, conductivity and the diamond crystal quality to further provide an efficient way to grow high-quality p-diamond material, restoring its lattice mismatch and demonstrating potential for p-BDD-based applications in optoelectronics.

## 4. Diamond-Based 1D Metal Oxide Heterojunction Classes

The combination of metal oxide and BDD has attracted wide attention, the main reason for which is the lack of effective n-type doping in diamond and p-type doping in metal oxide. The configuration of p-BDD and n-metal oxide has been most widely studied. In contrast to a continuous uniform film [26,27,32,65,66,67,68,69,70,71,72,73], the 1D nanostructure (e.g., NRs, NWs and NTs) that is free of defects, quantum-enhanced and has a large surface-area-to-volume ratio will not encounter the thermal mismatch with diamond, thereby substantially improving the performance of the hybrid structural heterojunction. For simplicity, we will focus on three different 1D n-type metal oxide (ZnO, TiO_2_ and WO_3_)/p-BDD heterojunction structure types in this review in the following general examples.

### 4.1. D N-ZnO/P-Diamond Heterojunction

ZnO is a suitable unintentionally doped n-type semiconductor with the wide band gap of 3.7 eV and exciton binding energy as high as 60 meV, which is promising for a wide range of applications. Various forms of ZnO nanostructures, such as NWs [15], NRs [74], nanobelts [75] and nanosheets [76], have been commonly studied because of their favorable optoelectronic performance compared with the bulk material. In these nanomaterials mentioned above, 1D n-ZnO nanostructures are supposed to be the most efficient for optoelectronic diodes owing to their high-density surface trap states, fewer interference states, excellent carrier confinement and grain boundaries [77]. They are able to improve the optoelectronic performance of photodiode devices [6,78].

#### 4.1.1. Epitaxial Growth of 1D ZnO NRs/Diamond Facet

**Figure 7:**
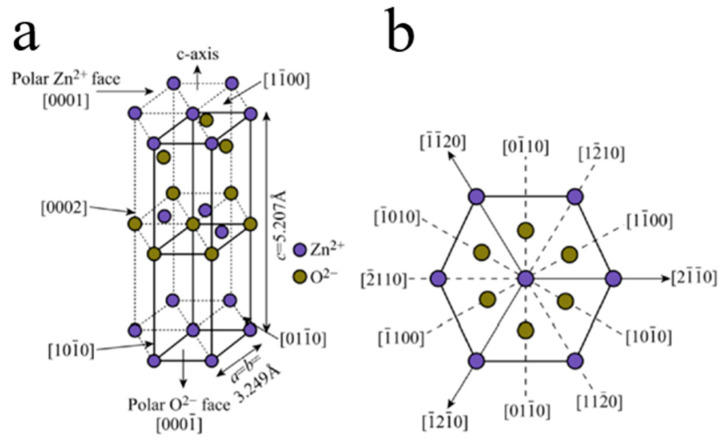
(**a**) ZnO unit cell with the hexagonal wurtzite structure. (**b**) Multiple crystal facets of the ZnO hexagonal wurtzite [79].

At room temperature and normal pressure, ZnO shows the hexagonal wurtzite structure (Figure 7). Each Zn^2+^ ion is surrounded with four O^2−^ ions corresponding to tetrahedral coordination (*sp*^3^ orbital hybridization) corresponding to the C_6V_^4^ class and *P*6_3_*mc* space group. The shape of Zn^2+^-O^2−^ tetrahedrons, as well as the ionic–covalent nature of Zn-O bonding, define the intrinsic polarity in the ZnO crystal growing along the c-axis [0001] orientation. Therefore, various 1D nanostructures are readily formed in the ZnO growth process [79].

**Figure 8:**
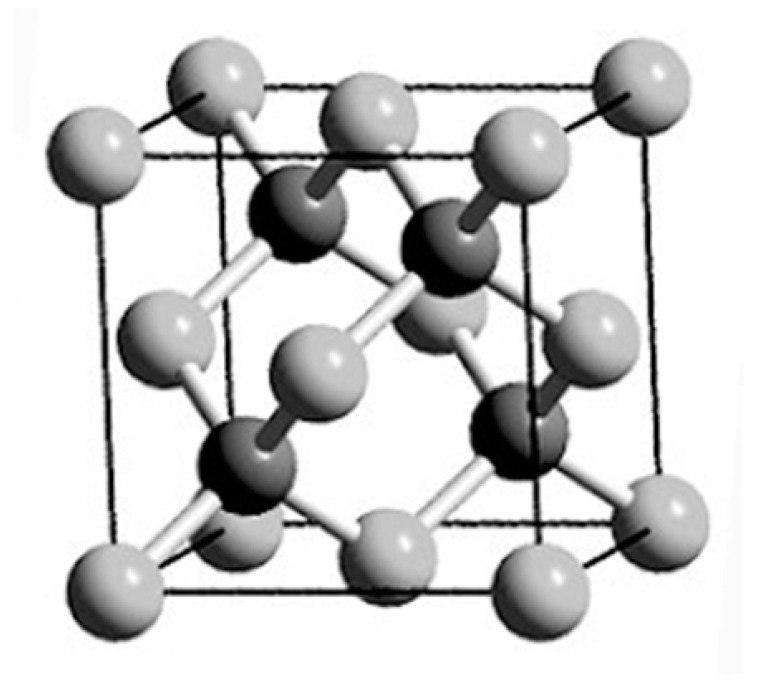
Diamond cell with a face-centered cubic structure [80].

Diamond is a typical atomic crystal with a face-centered cubic structure, belonging to the equiaxed crystal system. The carbon atoms in the diamond structure are bonded by *sp*^3^, with each carbon atom being bonded with four adjacent carbon atoms. The arrangement of diamond carbon atoms is shown in Figure 8, which contains a carbon atom centered in a regular tetrahedron and a carbon atom at each of four vertices, wherein carbon atoms at each vertex are covalently bonded with C-C bonds and shared by four tetrahedrons to form a three-dimensional network of diamond crystal [80].

**Figure 9:**
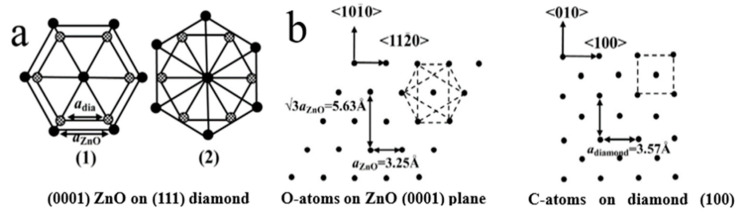
Schematic illustration atomic arrangement of (**a**) ZnO (0001) and diamond (111) planes and (**b**) ZnO (0001) facet and diamond (100) plane epitaxial relationship [23].

In terms of theoretical background, Li’s group proposed the epitaxial growth mechanism for the structure of ZnO-diamond. Since both ZnO crystal (0001) facet and diamond (111) facet have the same hexagonal atomic arrangement, ZnO (0001) planes and diamond (111) are geometrically matched. The [0001] oriented 1D ZnO NRs are normally aligned and epitaxial grown perpendicular to the diamond (111) plane. The epitaxial growth relation between the ZnO (0001) and diamond (111) is suggested in compliance with (0001) [11$\bar{2}$0]ZnO//(111)[1$\bar{1}$0] diamond or (0001)[10$\bar{1}$0]ZnO//(111) [1$\bar{1}$0]diamond (Figure 9a), and the epitaxial growth relation between the (0001) ZnO and (100) diamond is primarily of (0001)[0001]ZnO//(101) [101] diamond (Figure 9b) [23]. An in-depth study of 1D ZnO/diamond system will not only contribute to understanding the physical mechanism for epitaxial growth but also to extending the area of ZnO/diamond applications in optoelectronics devices.

#### 4.1.2. 1 D N-ZnO/P-Diamond Related Optoelectronic Devices

**Figure 10:**
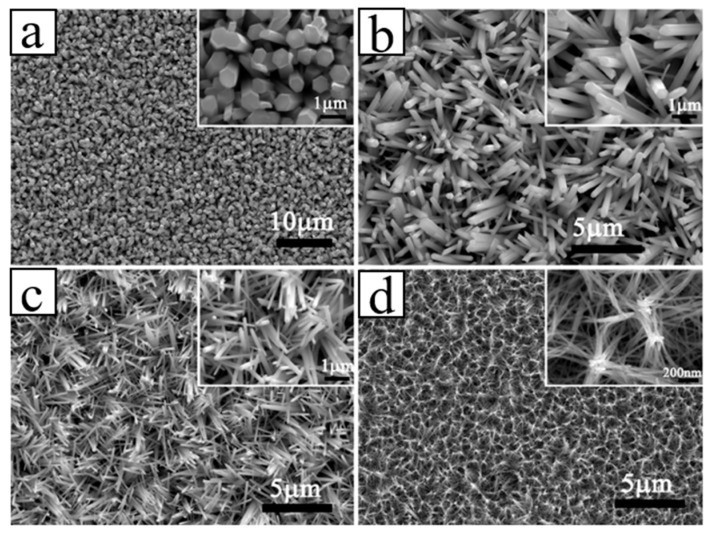
SEM micrographs with different morphologies (the insets) of the fabricated ZnO NRs based on various reaction concentrations: (**a**) 0.1, (**b**) 0.05, (**c**) 0.025 and (**d**) 0.01 mol/L [82].

**Figure 11:**
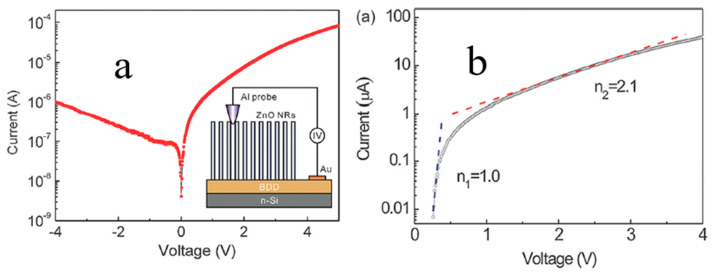
(**a**) The *I-V* curve of ZnO nanorod (NR)/BDD heterojunction. Insert shows the schematic configuration for conducting measurements. (**b**) The forward-biased ln *I-V* curve of ZnO NR/BDD heterojunction. A diode ideality factor *n*_1_ = 1.0 in the range from 0–0.3 V and *n*_2_ = 2.1 in the range from 1.2–3.0 V were simulated [82].

In the past, the existing ZnO types were usually deposited on a diamond substrate in the form of thin-film, which was used for surface acoustic wave (SAW) filters applications [65], examining structural and electrical properties [66,67,72], and for constructing p-n junction diode [68]. In the last ten years, composite 1D ZnO related-nanostructures and diamond nano-optoelectronic p-n junction devices were extensively studied. Zhi et al. [69] developed novel tyrosine biosensor with the biological function based on ZnO NRs on the BDD substrates. The ZnO NRs were for the first time deposited on BDD thin film by a low-temperature solution method. Boron-doped ZnO nanorods were fabricated on the BDD by hydrothermal technique and showed high photocatalytic performance [33]. Gao et al. [81] reported on photocatalytic activity of the n-ZnO NRs/p-BDD heterojunction fabricated with the hydrothermal degradation of methyl orange dye, which depended on the density and diameter of ZnO NRs, the distance between NRs and the interface at ZnO-BDD heterojunction (Figure 10). Vertically aligned ZnO NRs were synthesized by Wang et al. [82] for the first time by using a low-temperature solution method on the BDD films. The heterojunction showed typical rectification characteristics with the standard ideality factor approaching theoretical values under both low and high forward-biasing voltages (Figure 11).

**Figure 12:**
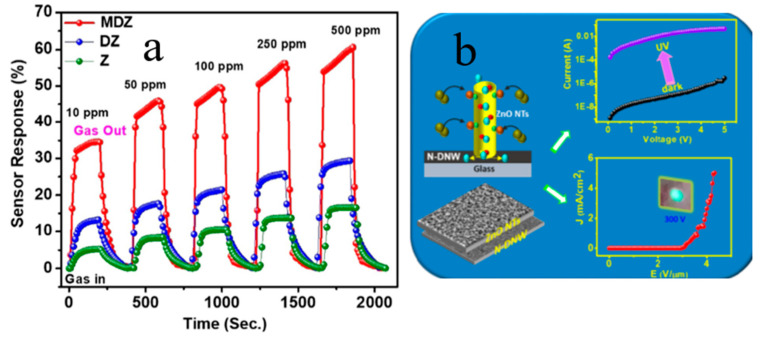
(**a**) Sensor response curve of pure ZnO NRs, ZnO NRs/ultra-nanocrystalline diamond, metallic glass/ultra-nanocrystalline diamond/ZnO NRs. (**b**) *I-V* characteristics and electron field emission properties of ZnO nanotubes incorporating diamond nanowires (NWs) in the dark and under UV illumination, respectively [85,86].

In recent years, ZnO NRs/BDD have been prepared and used as an anode for photocatalytic oxidation of aniline. The results showed that ZnO NRs/BDD is a promising photoanode for organic degradation [83]. Huang et al. [84] proposed a low-temperature annealing process to improve the optical response of the ZnO NRs/nano-diamond film substrate for the UV photodetector. Furthermore, novel ZnO NRs/ultra-nanocrystalline diamond may be used as a high-sensitivity device for hydrogen gas sensing (Figure 12) [85]. They also reported ZnO NTs, with the diamond NWs enhancing UV detection and field emission properties [86].

**Figure 13:**
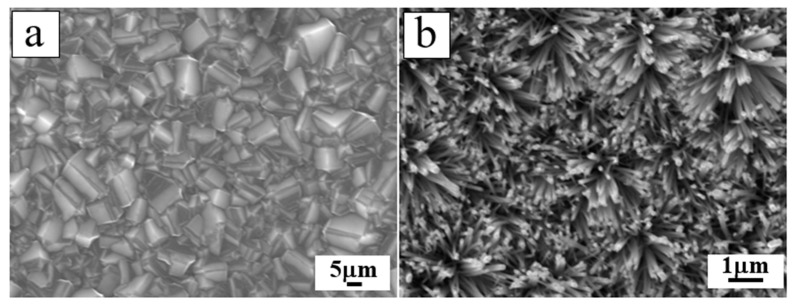
SEM morphology of (**a**) the plan view of the BDD film, (**b**) ZnO NWs deposited on the BDD film [30].

**Figure 14:**
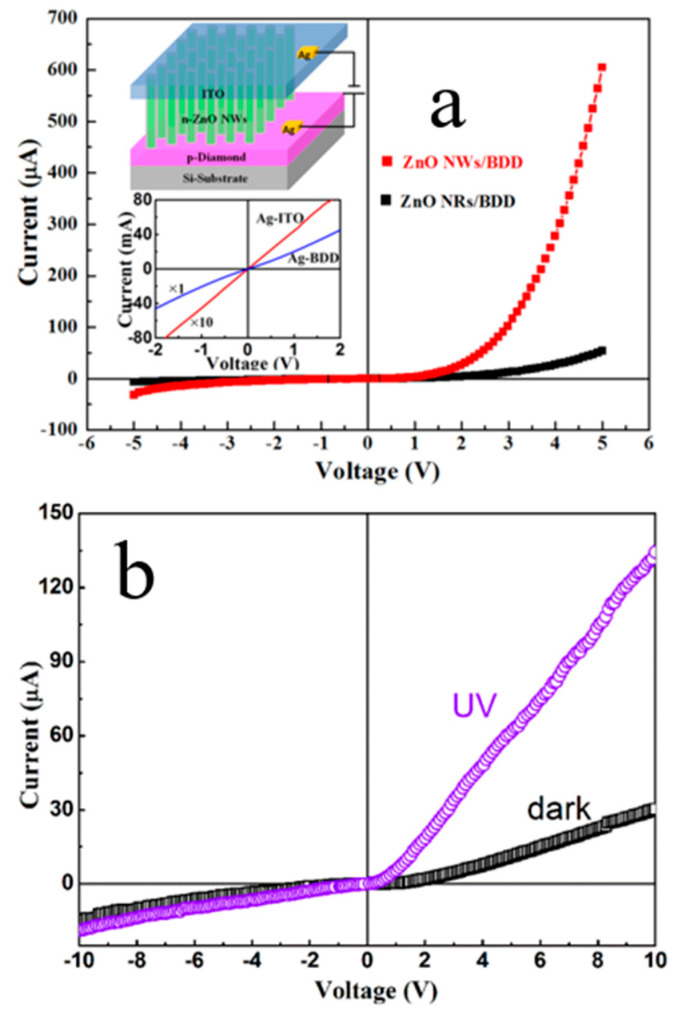
(**a**) *I-V* characteristics of the n-ZnO NWs/p-BDD and n-ZnO NRs/p-BDD heterojunctions, respectively. The top inset shows the schematic diagram of n-ZnO NWs/p-BDD heterojunction; the bottom inset shows ohmic contacts measurements for Ag/ZnO and Ag/BDD [30]. (**b**) *I-V* characteristics of n-ZnO NRs/p-BDD heterojunction with UV illumination and dark, respectively [22].

Compared to n-ZnO NRs/p-BDD heterojunction, n-ZnO NWs/p-BDD heterojunction reported by Sang et al. shows better *I-V* electrical properties and higher electrical transport performance (Figure 13 and Figure 14) [30]. By comparing both dark and UV illuminated *I-V* characteristics, one may see that the UV illumination reduces the turn-on voltage and ideality factor of the tested heterojunction. At 10 V, the UV positive current is more than 4 times as much as the dark [31]. To sum up, the high UV response rate, long device life and fast switching speeds of the heterojunction make it an ideal material for multifunctional optoelectronic applications.

#### 4.1.3. Electrical Transport Behavior of N-ZnO NRs/P-BDD Heterojunction at Elevated Temperatures

**Figure 15:**
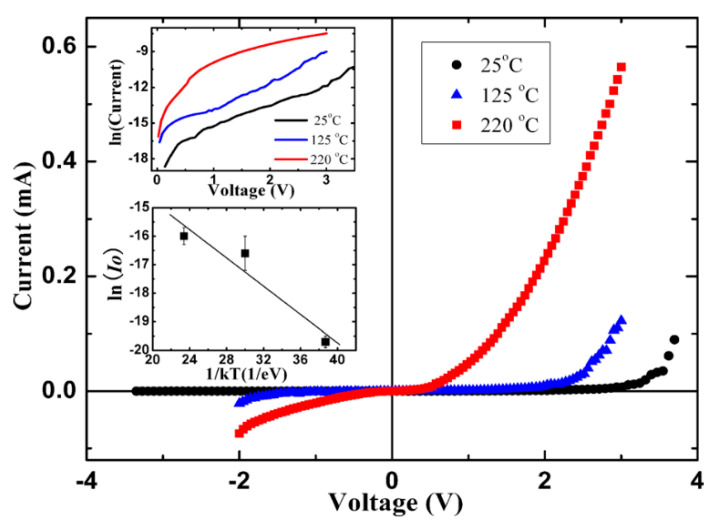
*I-V* plots of n-ZnO NRs/p-BDD heterojunction at the temperatures of 25 °C, 125 °C and 220 °C. The inset of the top presents the curve of the ln (*I*)-*V*. The bottom inset shows the ln (*I_S_*) vs. 1/*kT* curve [24].

Both diamond and ZnO are significant high-temperature materials, so the behavior of n-ZnO/p-BDD system is investigated at elevated temperatures. Sang et al. [24] reported on the preparation progress of n-ZnO NRs/p-BDD heterojunction devices and their electrical transport behavior in the temperature range from 25 °C to 220 °C. The device exhibits representative rectifying behavior at all test temperatures (Figure 15). The turn-on voltage of heterojunction decreases at higher temperatures. This is due to the fact that at higher temperatures, more holes are produced on the BDD side. The conductivity was lower than that of n-ZnO NRs/p-Si heterojunction [87], indicating that n-ZnO/p-BDD-based devices have better electrical properties at high temperatures. The decrease of *n* at higher temperatures is due to the increasing number of thermally excited carriers, the enhanced barrier tunneling, and the recombination process in the depletion region. At higher temperatures, both the carrier injection and ohmic behavior are improved. The detection temperature of n-ZnO NRs/p-BDD reaches about 220 °C, which is currently the maximum temperature for the p-n heterojunctions based on n-ZnO NRs.

#### 4.1.4. NDR for 1D N-ZnO/P-Degenerated BDD Heterojunction at Elevated Temperatures

**Figure 16:**
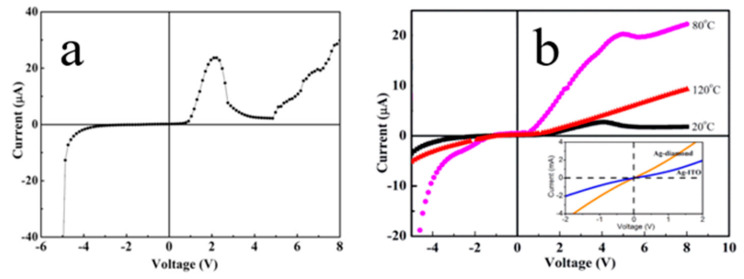
(**a**) *I-V* plots of the n-ZnO NR/p-BDD heterojunction fabricated with p-degenerated-diamond, displaying the NDR phenomenon. The inset shows a thermal equilibrium energy band diagram heterojunction. (**b**) The *I*-*V* curve of the n-ZnO NRs/p-degenerated diamond at 20~120 °C. The inset presents a linear relationship of *I*-*V* plots for ohmic contacts of Ag/p-degenerated diamond and Ag/ITO [23,31].

When p-diamond is degraded by heavy boron-doping (higher carrier concentration, 10^20^ orders of magnitude), the heterojunction exhibits the NDR phenomenon. The n-ZnO/p-degenerated BDD heterojunction provides a new way to form an NDR tunneling diode. The NDR phenomenon of 1D n-ZnO NRs/p-degenerate BDD was observed by Li et al. [23], and the forward *I-V* plots can be divided into three regions. The NDR phenomenon occurs with the peak-valley current ratios (PVCR) of ~10 (Figure 16a). The generation of NDR is caused by the tunneling current in the ZnO and p-degenerated BDD structures.

In our most recent study [31], we reported on n-ZnO NRs/p-degenerated BDD tunnel diode and its NDR characteristics with temperature change as well as carrier tunnel injection behavior. The results show that the prepared heterojunction exhibits NDR performance at 20 °C and 80 °C. Subsequently, NDR disappears, and rectifying properties become visible at 120 °C (Figure 16b). The PVCR is reduced at higher temperatures. The tunneling current from diamond valence states to ZnO deep levels leads to the reduction in the turn-on voltage at higher temperatures and promotes the appearance of NDR. The high-temperature NDR phenomenon of 1D n-ZnO NRs/p-degenerated BDD heterojunction may extend the resistance switches and resonant tunneling diode applications to high-temperature and high-power environments.

Table 1 provides an overview of a portion of recent investigations based on 1D n-ZnO/p-diamond heterojunction for various applications and properties.

### 4.2. 1 D N-TiO_2_/P-Diamond Related Optoelectronic Devices

1D TiO_2_ nanostructures with a wide bandgap (3.2 eV) and a large surface area [88] were selected as a promising material for application in electronics [89,90,91]. Common functional TiO_2_ nanomaterials have attracted widespread attention due to superior performance derived from their inherent 1D architecture [92,93,94]. 1D nanostructures offer more possibilities for better optical and electrical properties, such as faster carrier generation, better charge separation, and longer charge carrier life [95,96,97]. TiO_2_ is an intrinsic n-type semiconductor and may form a p-n heterojunction combined with the p-type semiconductor [98,99,100,101,102]. N-type TiO_2_ and p-type BDD have excellent and unique properties, which make them ideal semiconductors for forming heterojunction structures.

**Figure 17:**
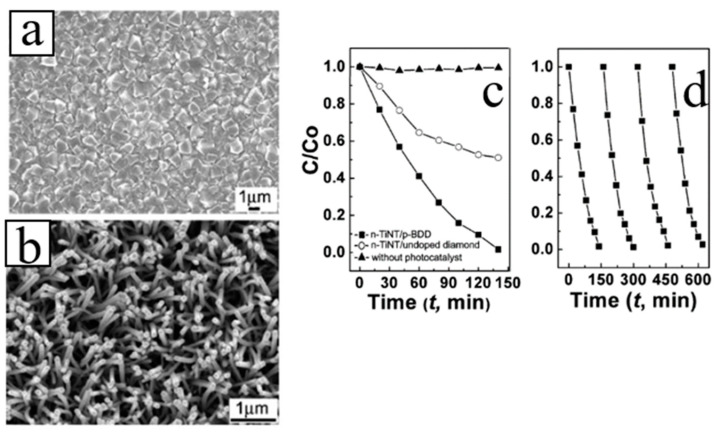
(**a**) SEM morphology of the BDD film; (**b**) plan-view SEM morphology of TiO_2_ NTs on BDD; (**c**) photocatalytic decomposition of RY15 solution of n-TiO_2_ NTs/p-BDD heterojunction, TiO_2_ NTs on the undoped diamond and without photocatalyst. (**d**) Recyclability test of photocatalytic decomposition for n-TiO_2_ NTs/p-BDD heterojunction [38,39].

**Figure 18:**
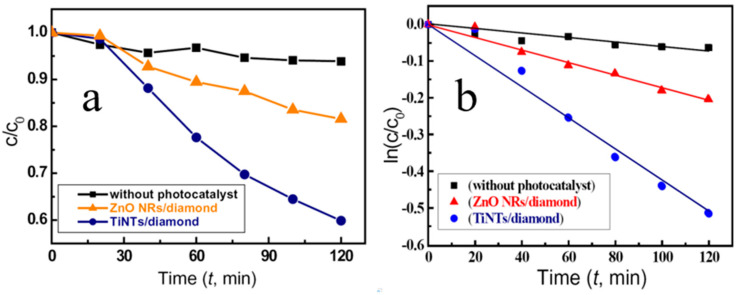
Photocatalytic decomposition of RY15 solution with (**a**) TiO_2_ NTs on hemispherical diamond film (TiO_2_ NTs/diamond). (**b**) The linear relationship between ln(C/C_0_) and time [104].

In their previous study, Li et al. [103] deposited 1D TiO_2_ NRs with different morphology grown on BDD film with ZnO layer using the hydrothermal method. TiO_2_ NRs/ZnO/BDD heterojunction may enhance the photocatalytic activity of TiO_2_ NRs/ZnO and TiO_2_ NRs/BDD heterojunctions, respectively. In addition, TiO_2_ NTs arrays were fabricated on the p-type BDD substrate by means of liquid phase deposition using ZnO NRs template. Compared with the case of a single TiO_2_ NTs, n-TiO_2_ NTs/p-diamond heterostructure shows significantly enhanced photocatalytic activity and good recyclability (Figure 17) [38,39]. Afterward, TiO_2_ NTs were deposited by the same method on hemispherical CVD diamond films. The degradation of reactive yellow 15 (RY15) solution showed that the product exhibits high photocatalytic performance (Figure 18) [104].

**Figure 19:**
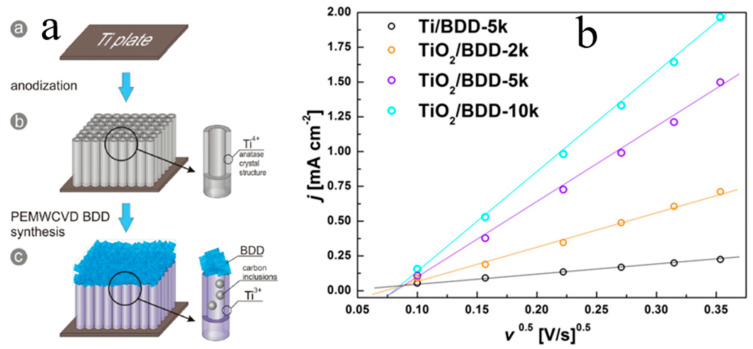
Schematic illustration for composite TiO_2_ NTs/BDD electrode. Cyclic voltammetry plots: (**a**) combination electrodes TiO_2_ NTs/BDD-10 k, with pure TiO_2_ and BDD immersed in 0.1 M NaNO_3_ (ν = 50 mV/s), (**b**) the curve of peak currents versus the square root of the scan rate for different BDD-covered electrodes [70,105,106].

TiO_2_ NTs/BDD composite electrode (Figure 19a) was fabricated by microwave plasma-enhanced chemical vapor deposition (CVD) by the Siuzdak group, which greatly enhanced the electrochemical properties. Compared with the BDD prepared on a Ti plate (0.11 mF·cm^−2^), the composite electrode provides high capacitance (Figure 19b). The enhanced capacitive effect in TiO_2_ NTs/BDD can be understood as follows: (1) the particular cooperative morphology of TiO_2_ NTs and BDD provides a more efficient conduction route for ion diffusion; (2) NTs are partially decomposed and converted to Ti_2_O_3_ and TiC fractions. Finally, TiO_2_ NTs, which demonstrate high ordering attained by a simple, rapid and controllable anodic oxidation method, can be used as the conductive BDD layer deposited on a substrate and then embedded into the structure of a supercapacitor [70,105,106].

**Figure 20:**
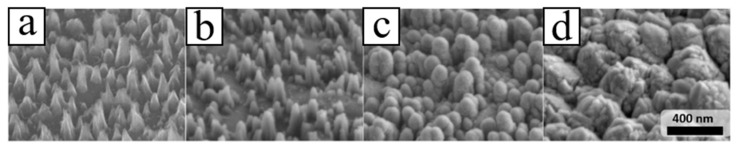
SEM morphology of BDD (**a**) BDD with 20 nm (**b**), 100 nm (**c**) and 500 nm (**d**), thick TiO_2_ layer [41].

Most recently, Miroslav et al. [41] reported on the development of a unique photoelectrochemical electrode consisting of a nanostructured BDD layer covering an n-type TiO_2_ film (Figure 20). The effects of the nanostructure, B doping level and TiO_2_ film thickness on the properties of PEC were studied. Using RF magnetron sputtering, the BDD films with two doping levels (gas phase B/C = 1000 ppm and 10,000 ppm) that had already grown and plasma-nanostructured were used as the substrates to deposit TiO_2_ layers of 20, 100 and 500 nm. BDD was used as the structure electrode. When the gas phase B/C ratio was 1000 ppm, the surface was covered with a TiO_2_ layer 500 nm thick, and the photocurrent was the highest (60 μA/cm^2^ and 3.2 mA/cm^2^ at 0 and 2 V vs. Ag/AgCl respectively). The favorable influence of the nanostructure type and pn junction on the hole injection was confirmed by experiments. Suzuki et al. [37] prepared a mesoporous TiO_2_ layer on the BDD and found that deep-UV illumination could improve its photocatalytic efficiency. They also used this hybrid electrode in water electrolysis to produce O_3_ gas and other reactive oxygen species (ROS). In addition, ROS produced by electrochemistry were tested in water treatment under UV illumination and demonstrated the system’s suitability for advanced oxidation processes [27]. This simple water treatment system can be used to break down refractory organic matter in wastewater, a process that is still being studied. Basically, the 1D n-TiO_2_/p-BDD device suggests a new strategy, which is expected to be used for its wide-range implementation in photocatalysis, photoelectric chemical electrode and sensor, etc.

A summary of different synthesis routes and applications (properties) of 1D n-TiO_2_/p-diamond heterojunction is presented in Table 2.

### 4.3. 1D N- WO_3_/P-BDD-Related Optoelectronic Devices

WO_3_ is a low-cost metal oxide semiconductor with a wide band gap (2.7 eV), excellent electron transport and unintentionally n-type doping performance. WO_3_ is considered as another candidate material for applications in electronics [7,107]. 1D WO_3_ nanostructures (such as NRs, NWs and nanoneedles) have been used in photocatalytic applications [108,109], sensor switching devices [110], gas sensors [111,112] and UV photodetectors [2]. Because of the excellent performance of 1D WO_3_ and BDD, it is worth combining them in 1D n-WO_3_/p-BDD heterojunction to provide new applications in electronics, especially at higher temperatures.

**Figure 21:**
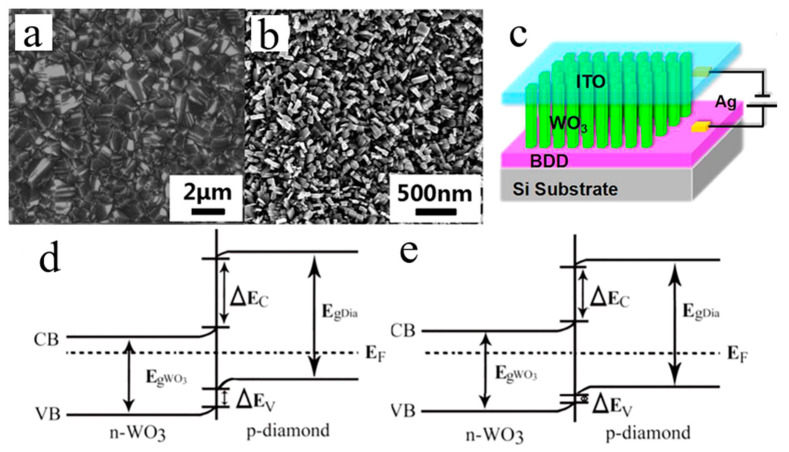
(**a**) SEM morphology of the BDD substrate and (**b**) WO_3_ NRs on the BDD substrate. (**c**) Schematic diagram of the n-WO_3_ NRs/p-BDD heterojunction. (**d**) Room temperature and (**e**) higher-temperature energy band diagrams of the n-WO_3_ NRs/p-BDD heterojunction [34].

**Figure 22:**
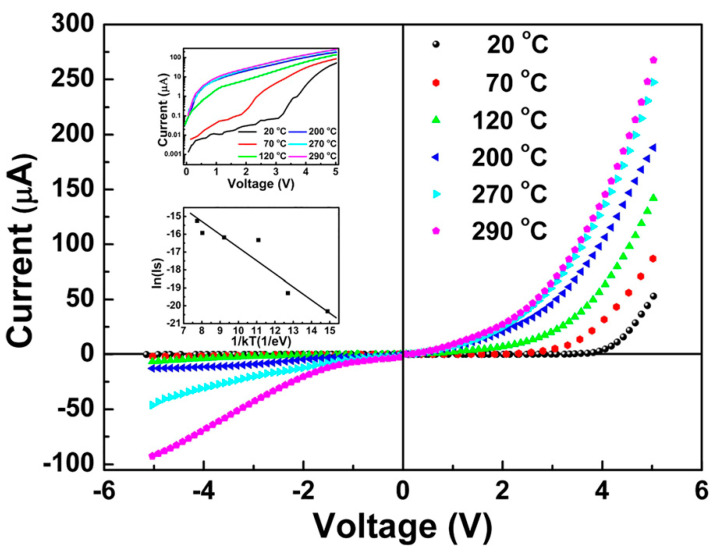
*I-V* curve of the n-WO_3_ NRs/p-BDD heterojunction operating from 20 °C to 290 °C. The top inset is the curve of log *I*-log *V*, and the bottom inset is the relation of ln (*I_s_*) vs. 1/*k_B_T* [34].

In 2017, Li et al. [34] studied the high-temperature electrical transport behavior of n-WO_3_ NRs/p-BDD heterojunctions fabricated by the hydrothermal method. WO_3_ NRs with a square feature of the top surface were deposited onto the BDD with perpendicular alignment to the substrate plane (Figure 21a–c). The p-n heterojunction demonstrated excellent thermal stability and rectified characteristics within the temperature range from room temperature to 290 °C. The turn-on voltages declined, and the rectified ratio was relatively high with the temperature increase. The ideality factor decreases with the increase in temperature (Figure 21b). Under the influence of high temperature, when the reverse voltage is large, both the forward current and the reverse leakage current increase (Figure 22).

At elevated temperatures, the E_F_ value of the heterojunction semiconductor usually moves close to the middle region of the bandgaps (shown in the energy band diagrams, Figure 21d,e) [113]. More intrinsic carriers are thermally excited, and it becomes easier for electrons (holes) to pass from the n-WO_3_ conduction band (p-BDD valence band) to the p-BDD conduction band (n-WO_3_ valence band) under the voltage applied, which leads to the decreasing turn-on voltage, increasing forward current, and slightly improved injection efficiency. At higher voltages and for temperatures above 200 °C, the traps are filled with more thermally excited carriers, and the injected current complies with the trap-free SCLC law, with an exponential power of about 2. The E_t_ value of the characteristic trap energy obtained from the heterojunction is 50 meV (inset of Figure 22). The characteristic trap energy values measured for ZnO NRs/BDD heterojunction [24], amorphous polymer semiconductor [114] and organic heterojunction [115] are higher than the listed value.

Compared with the previously reported heterojunctions comprising WO_3_ (n-WO_3_/p-carbon NTs [116] and WO_3_/SnO_2_ [117]), the structure of 1D n-WO_3_/p-BDD has better electrical transport performance at higher temperatures. This study extends the ranges of the design and application for heterojunctions based on BDD, especially their employment at high temperatures, high power and in various harsh environments.

## 5. Conclusions and Future Perspective

In this review, we have summarized recent general findings for diamond-based metal oxide p-n heterojunctions, especially those that comprise three important types of 1D nanostructures studied in this photoelectric decade. The features of p-type BDD films and corresponding epitaxial growth relationships of 1D ZnO NRs on diamond facet have been experimentally and theoretically predicted. High-temperature electrical transport behavior and NDR of n-ZnO NRs/p-BDD heterojunction have been presented in detail. 1D n-TiO_2_/p-BDD heterojunction-related optoelectronic applications, including photocatalysis and photoelectrochemical electrode, have been presented. The fabrication of 1D n-WO_3_/p-BDD heterojunction and its electronic behavior at high temperature are discussed.

These above-mentioned reports provide a greatly expanded understanding of the background of electrical properties in diamond-based 1D metal oxides p–n heterojunctions. In the future, it is also desirable to find new fabrication routes and types of electrodes for a device to improve the device performance. For instance, by introducing a doping element into the metal oxide, one may increase the carrier concentration or fabricate composite 1D metal oxide with other nanomaterials to form complex structures. Optimization of the growth quality for BDD will raise the operating temperature of the device. By changing the electrode configuration of the heterojunction and controlling highly oriented nanosized 1D metal oxide and boron doping concentration of the BDD, we could construct a variety of heterojunction devices and optimize the preparation process. In order to better understanding the device tunneling physical mechanism, it becomes essential to simulate semiconductor theoretical and computational models for the development of diamond-based metal oxides p-n heterojunctions. This is not a comprehensive investigation, but rather a report on the most cited and unique explanations that look promising for the future. These may be only a small part of the possibilities, and the authors hope that this review will inspire further research activities to make more discoveries in the future.

## Figures and Tables

**Table 1:** Heterojunction applications and properties of 1D n-ZnO/p-diamond.

Heterojunction Morphology	Synthesis Route	Applications (Properties)	Ref.
ZnO film/diamond	Magnetron sputtering	Surface acoustic wave	[65]
ZnO film/diamond	Magnetron sputtering	Films’ quality	[66]
ZnO film/diamond	MOCVD	Electrical properties	[67]
ZnO film/diamond	Atomic Layer Chemical Vapour Deposition	Electrical properties	[72]
ZnO film/diamond	Magnetron Sputtering	Heterojunction diode	[68]
ZnO NRs/diamond	Low-temperature solution	Tyrosinase biosensor	[69]
ZnO NRs/diamond	Hydrothermal method	Photocatalytic activities	[33]
ZnO NRs/diamond	Hydrothermal method	Photocatalytic activities	[81]
ZnO NRs/diamond	Low-temperature solution	Electrical properties	[82]
ZnO NRs/diamond	Hydrothermal method	Photoelectric anodes	[83]
ZnO NRs/diamond	Hydrothermal method	UV photodetector	[84]
ZnO NRs/diamond	Sol-gel method	Hydrogen gas sensors	[85]
ZnO NTs/diamond	Hydrothermal method	UV detection and field emission	[86]
ZnO NWs/diamond	Hydrothermal method	Electrical transport properties	[30]
ZnO NRs/diamond	Thermal evaporation method	NDR properties	[31]
ZnO NRs/diamond	Thermal evaporation method	UV photoelectrical properties	[22]
ZnO NRs/diamond	Thermal evaporation method	Electrical transport behavior	[24]
ZnO NRs/diamond	Thermal evaporation method	NDR properties	[23]

**Table 2:** Heterojunction applications and properties of 1D n-TiO_2_/p-diamond.

Heterojunction Morphology	Synthesis Route	Applications (Properties)	Ref.
TiO_2_ film/diamond	Sol-gel method	Photoelectrocatalytic activities	[102]
TiO_2_ NRs/diamond	Hydrothermal method	Photocatalytic activities	[103]
TiO_2_ NTs/diamond	Liquid phase deposition method	Photocatalytic activities	[38]
TiO_2_ NTs/diamond	Liquid phase deposition method	Photoelectronic nanodevices	[39]
TiO_2_ NTs/diamond	Liquid phase deposition method	Photocatalytic devices	[104]
TiO_2_ NTs/diamond	Anodization method	Hybrid electrode	[105]
TiO_2_ NTs/diamond	Anodization method	Supercapacitor or Energy Storage Devices	[106]
TiO_2_ NTs/diamond	Anodization method	Supercapacitor	[70]
TiO_2_ film/diamond	Radio Frequency sputtering	Photoelectrochemical performance	[41]
TiO_2_ film/diamond	Sol-gel method	Photocatalytic activities	[37]
TiO_2_ film/diamond	Sol-gel method	Hybrid electrode	[27]
